# Study on the causes of changes in colour during *Hibiscus syriacus* flowering based on transcriptome and metabolome analyses

**DOI:** 10.1186/s12870-024-05142-0

**Published:** 2024-05-21

**Authors:** Zhezhe Li, Dan Liu, Dongsheng Wang, Meng Sun, Guojun Zhang, Yu Wu, Yidan Zhang, Beibei Cheng

**Affiliations:** 1 Hebei Normal University of Science & Technology, Qinhuangdao, 066004 Hebei Province China; 2 Hebei Key Laboratory of Horticultural Germplasm Excavation and Innovative Utilization, Qinghuangdao, 066004 Hebe Province China; 3 Shandong Provincial Forest and Grass Germplasm Resources Center, Jinan, 250102 Shangdong Province China; 4 Hebei Higher Institute Application Technology Research and Development Center of Horticultural Plant Biological Breeding, Hebei Normal University of Science & Technology, Qinhuangdao Hebei Province, Qinhuangdao, 066004 Hebe Province China

**Keywords:** *Hibiscus syriacus*, Transcriptome sequencing, Flower colour change, Flavonoid

## Abstract

**Background:**

The flower colour of *H. syriacus* ‘Qiansiban’ transitions from fuchsia to pink–purple and finally to pale purple, thereby enhancing the ornamental value of the cultivars. However, the molecular mechanism underlying this change in flower colour in *H. syriacus has not been elucidated*. In this study, the transcriptomic data of *H. syriacus* ‘Qiansiban’ at five developmental stages were analysed to investigate the impact of flavonoid components on flower colour variation. Additionally, five cDNA libraries were constructed from *H. syriacus* ‘Qiansiban’ during critical blooming stages, and the transcriptomes were sequenced to investigate the molecular mechanisms underlying changes in flower colouration.

**Results:**

High-performance liquid chromatography‒mass spectrometry detected five anthocyanins in *H. syriacus* ‘Qiansiban’, with malvaccin-3-*O*-glucoside being the predominant compound in the flowers of *H. syriacus* at different stages, followed by petunigenin-3-*O*-glucoside. The levels of these five anthocyanins exhibited gradual declines throughout the flowering process. In terms of the composition and profile of flavonoids and flavonols, a total of seven flavonoids were identified: quercetin-3-glucoside, luteolin-7-*O*-glucoside, Santianol-7-*O*-glucoside, kaempferol-*O*-hexosyl-C-hexarbonoside, apigenin-C-diglucoside, luteolin-3,7-diglucoside, and apigenin-7-*O*-rutinoside. A total of 2,702 DEGs were identified based on the selected reference genome. Based on the enrichment analysis of differentially expressed genes, we identified 9 structural genes (*PAL*, *CHS*, *FLS*, *DRF*, *ANS*, *CHI*, *F3H*, *F3’5’H*, and *UFGT*) and 7 transcription factors (3 *MYB*, 4 *bHLH*) associated with flavonoid biosynthesis. The qRT‒PCR results were in good agreement with the high-throughput sequencing data.

**Conclusion:**

This study will establish a fundamental basis for elucidating the mechanisms underlying alterations in the flower pigmentation of *H. syriacus*.

**Supplementary Information:**

The online version contains supplementary material available at 10.1186/s12870-024-05142-0.

## Introduction

*Hibiscus syriacus* L., a perennial deciduous shrub or small tree, belongs to the Malvaceae family and the genus *Hibiscus*. Its cultivars are rich in colour and include red, pink, purple, blue, and white. The trees are fully flowered in full bloom and have high ornamental value. The flowering period of individual *H. syriacus* is short and lasts only one day, a phenomenon also known as blooming in the morning and falling in the evening. Although the flowers of these plants have a short lifespan, the flower colour changes in some cultivars, such as ‘Qiansiban’, during the flowering process from fuchsia to pink‒purple and then to pale purple. This characteristic confers high ornamental and research value to this cultivar. Many plants change flower colour during flowering. Paeonia ‘Coral Sunset’ and ‘Pink Hawaiian Coral’ flowers change from coral to pink and then to pale yellow during flowering [1]. The corolla of purple‒red *Mirabilis jalapa* changes from light green to purple‒red between budding and blooming [2].

Flower colour, an important ornamental trait of plants, can be affected by anthocyanin content, cell sap pH, and metal ions. Among these, the anthocyanin components and contents are the most important factors. There are three main types of pigments related to flower colour formation: flavonoids [3], carotenoids [4], and alkaloids [5]. Flavonoids include mainly anthocyanins, flavones, and flavonols [6]. At present, 27 categories of anthocyanins have been identified in nature, of which only 6 are the most common, namely, cyanidin, geranium, delphinidin, paeoniflorin, petunia, and mallow [5]. Geranium is usually orange to red, cyanidin is pink, delphinidin is blue to purple, and flavonoids and flavonols are colourless or light yellow, which generally affects the formation of flower colour as pigments [7]. P Z et al. [8] detected major anthocyanins, including cyanidin, geranium, delphinidin, paeoniflorin, and petunia, in *H. syriacus*. In addition, the flavonoid biosynthetic pathway was studied in detail. In the flavonoid metabolism pathway, structural genes such as *CHS*, *CHI*, and *DRF* regulate the synthesis of anthocyanins and affect the colouration of *H. syriacus* [9].

However, the mechanism underlying the colour change in *H. syriacus* during flowering has not been determined. To investigate the underlying mechanism, *H. syriacus* ‘Qiansiban’, which changes flower colour during flowering, was selected as the experimental material. In the present study, flavonoids in petals at five developmental stages were identified via high-performance liquid chromatography‒mass spectrometry (HPLC‒MS), and the relationship between flower colouration and pigment content was analysed. Furthermore, a comparative transcriptomic analysis of five critical flowering states in ‘Qiansiban’ was conducted to identify the differentially expressed genes (DEGs) related to flavonoid biosynthesis. This research will help elucidate the mechanism of flower colour change in *H. syriacu*s.

## Materials and methods

### Plant materials

The petals of ‘Qiansiban’ were used as experimental materials. Samples were collected at five different flowering stages (S1, S2, S3, S4, S4, and S5) in July 2021 at the Hebei Normal University of Science and Technology, and 15 samples were collected at each stage. Materials for the tests were immediately placed in liquid nitrogen and stored at −80 °C.

### Anthocyanin quantification

Flower petals were collected at five sequential developmental stages: the splitting stage (S1), the early stage (S2), the initial opening period (S3), the blooming period (S4), and the final flowering stage (S5). A total of 0.1 g of *H. syriacu*s corolla from each of the five periods was ground into powder with liquid nitrogen, poured into a centrifuge tube, and soaked in 5 mL of a 1% hydrochloric acid–methanol solution in a dark refrigerator at 4 °C for 24 h (during several electric shocks), and the supernatant obtained after centrifugation at 4 °C for 15 min (13,000 r·min^− 1^) was submitted for determination by HPLC‒MS.

The samples were analysed with an HPLC‒MS system. The analysis methods used were described by C Y [10] and H C [11] and were slightly modified. The liquid-phase conditions were as follows: the column temperature was set at 40 °C, and 10.0 µL of sample was injected at 0.50 mL/min. An ultrapure aqueous solution (with 1% formic acid) was used as mobile phase A, and acetonitrile was used as mobile phase B. After 95% A + 5% B elution for 0 min, the volume fraction of B was increased to 40%, and the mixture was eluted for 40 min. Finally, the volume fraction of B was reduced to 5%, and the mixture was eluted for 40 min. The absorption spectra were scanned at 350 and 520 nm.

The MS conditions for the positive ion mode, electrospray ionization source, dryer, and ion source temperature were set at 400 °C and 120 °C, respectively. The capillary pyrolysis and cone hole voltages are 3.5 kV and 40 V, respectively. The scanning points were in the range of 100–1200 m/z.

With standard cyanidin and rutin as the controls, the concentration as the abscissa, and the peak area as the ordinate, a standard curve was generated to calculate the contents of anthocyanins and flavonoids.

The standard equation for cyanidin content was y = 528.57x−9.6762 (R2 = 0.9989); the standard equation for rutin content was y = 534.58x + 499.02 (R2 = 0.996).

### RNA extraction, cDNA library construction, and transcriptome sequencing

Total RNA was extracted using an RNAprep Pure Plant Kit (Tiangen, Beijing, China) according to the manufacturer’s instructions. Transcriptome sequencing and library construction were completed by BMK Biotechnology Co., Ltd. (China). First, mRNA was purified from total RNA using poly-T oligo-attached magnetic beads. First-strand cDNA was synthesized, and second-strand cDNA was subsequently generated. The remaining overhangs were converted into blunt ends via exonuclease/polymerase activities. After adenylation of the 3’ ends of the DNA fragments, an NEBNext Adaptor with a hairpin loop structure was ligated to prepare the fragments for hybridization. The library fragments were purified with the AMPure XP system (Beckman Coulter, Beverly, USA). Then, 3 µl of USER Enzyme (NEB, USA) was incubated with size-selected, adaptor-ligated cDNA at 37°C for 15 min followed by 5 min at 95°C before PCR. PCR was subsequently performed with Phusion High-Fidelity DNA polymerase, universal PCR primers, and Index (X) Primer. Finally, the PCR products were purified (AMPure XP system), and the library quality was assessed on an Agilent Bioanalyzer 2100 system. The transcriptome library of *H. syriacu*s ‘Qiansiban’ was subsequently sequenced on the Illumina NovaSeq 6000 sequencing platform.

### De novo assembly and annotation

The raw reads were filtered by removing adaptor and low-quality sequences to obtain high-quality reads. The gene functions were annotated based on the following databases: Nr (NCBI nonredundant protein sequences); Pfam (protein family); KOG/COG (Clusters of Orthologous Groups of proteins); Swiss-Prot (a manually annotated and reviewed protein sequence database); KO (KEGG Orthologue database); and GO (Gene Ontology).

### Verification by qRT‒PCR

The expression levels of key genes in the petals of ‘Qiansiban’ were measured using qRT‒qPCR. GAPDH was used as an internal reference gene for real-time fluorescence-based quantitative reactions. The 10 µl reaction mixture was formulated based on the instructions provided for the TB Green TaKaRa Premix Ex Taq™ II (Tli RNaseH Plus; TaKaRa Bio, Inc., Japan). The reaction procedure involved predenaturation at 95 °C for 30 s. The cycle stage was as follows: denaturation at 95 °C for 5 s and annealing at 60 °C for 30 s for 40 cycles. Dissolution curves were recorded from 60 °C to 95 °C with a 0.5 °C increase every 1 min. Each reaction was repeated three times. The relative expression levels of the target genes were calculated by the 2 − ΔΔCt method [12], and the relevant data were analysed with SPSS version 26.

## Results

### Qualitative and quantitative analyses of anthocyanins

**Fig. 1:**
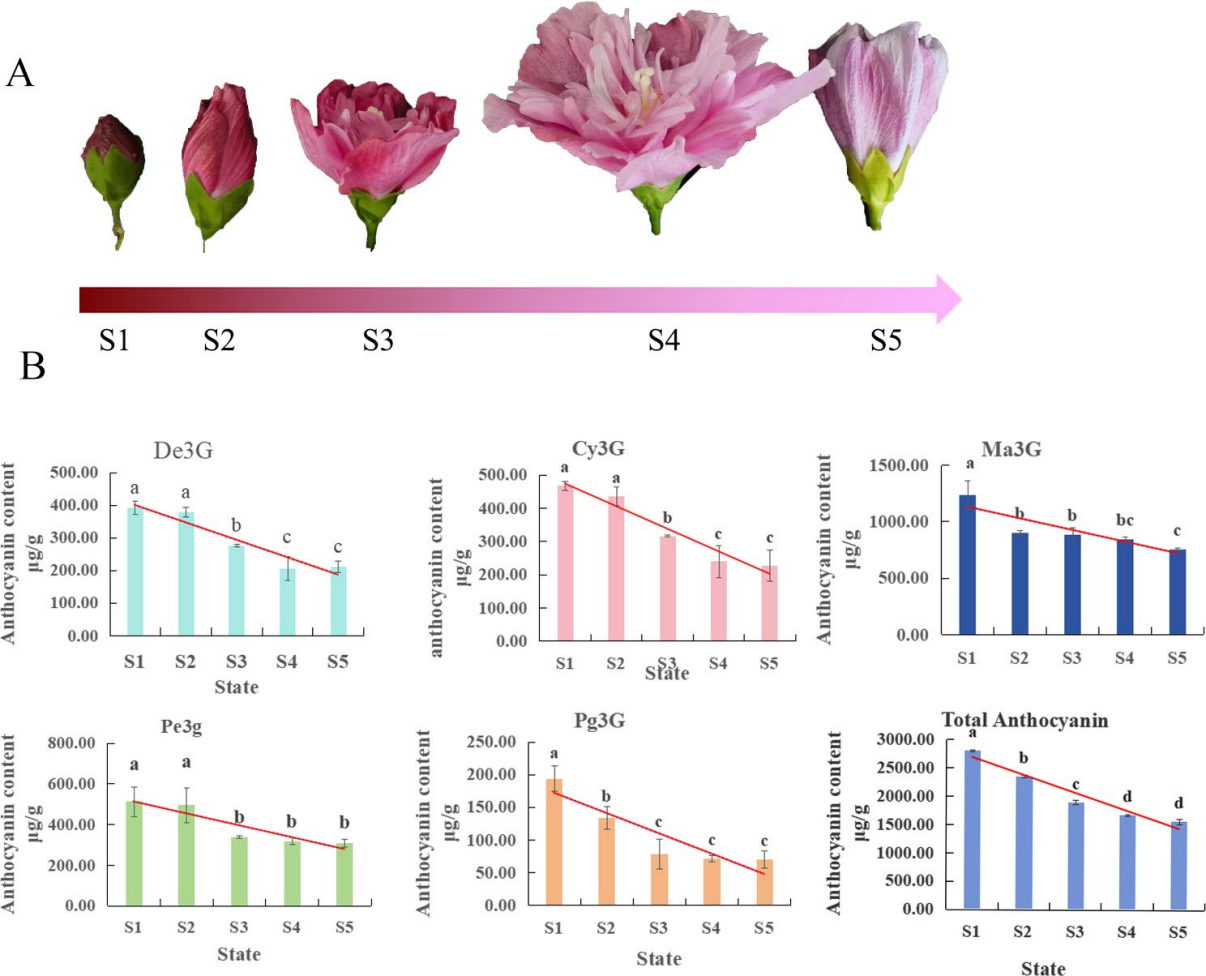
Anthocyanin contents in the ‘Qiansiban’ petals at different stages. (**A**) The colour of the flower samples varied from S1 to S5. (**B**) Total contents of individual anthocyanidins from S1 to S5. The bars represent the average data of the three HPLC‒MS/MS analyses

The flower colour of ‘Qiansiban’ is purplish red at the S1 stage, and as the flowers bloom, the colour gradually changes to pinkish purple and purple (Fig. 1A).

Anthocyanins and flavones were identified by comparison with the HPLC retention times, elution orders, UV–vis spectra, and MS fragmentation patterns in published data (Table 1). Through the analysis of molecular ions and fragment ions via mass spectrometry and in combination with previous reports [13–15], five anthocyanins were detected in the petals of ‘Qiansiban’ in the five stages, namely, delphinidin-3-*O*-glucoside (De3G), cyanidin-3-*O*-glucoside (Cy3G), petunidin-3-*O*-glucoside (Pe3G), pelargonidin-3-*O*-glucoside (Pg3G), and malvacin-3-*O*-glucoside (Ma3G) (Table 1). The remaining 7 compounds were speculated to be quercetin-3-glucoside, luteolin-7-*O*-glucoside, santianol-7-*O*-glucoside, kaempferol-*O*-hexose-C-hexoside, apigenin-C-glucoside, luteolin-3,7-diglucoside, and apigenin-7-*O*-rutoside (Table 1) [10, 11, 16].

*H. syriacus* petals undergo major changes in flavonoid and anthocyanin accumulation from S1 to S5. Anthocyanidin is the main contributor to the colour of *H. syriacus*; thus, its type, content, and biosynthesis process in *H. syriacus* petals were analysed thoroughly.

The flavonoid accumulation trend was S1 > S2 > S3 > S4 > S5, which gradually decreased with increasing flower opening. The analysis based on HPLC‒MS/MS revealed that all anthocyanidins were highest in content in S1 and lowest in content in S5. The contents of these five anthocyanins gradually decreased over the course of the flowering process, decreasing by 46%, 52%, 40%, 64%, and 39%, from S1 to S5, respectively. The anthocyanidin with the highest content in S1 was Ma3G (Fig. 1B), and the flavone with the highest content was apigenin-C-glucoside (Supplementary Table S1).

In summary, the accumulation of flavonoids and anthocyanidin reached the highest and lowest levels, respectively, at S1 and S5. Ma3G (the anthocyanidin with the highest content), De3G, Pe3G, Cy3G, and Pg3G were the main chromogenic anthocyanidins. With decreasing anthocyanidin concentration, the colour of the petals changed from purplish red to purple (Fig. 1).

**Table 1:** Chromatographic and spectral data of flavonoids from ‘Qiansiban’

Peak	Retention time (min)	Molecular mass	Fragmentation	Tentative identification
1	11.4	465.1	303.05	Delphinidin-3-*O*-glucoside
2	13.2	449.1	287.05	Cyanidin-3-*O*-glucoside
3	13.95	479.11	317.06	Petunidin-3-*O*-glucoside
4	14.84	433.11	271.06	Pelargonidin-3-*O*-glucoside
5	16.3	493.13	331.08	Malvacin-3-*O*-glucoside
6	11.28	465.1	303.05	Quercetin-3-glucoside
7	13.07	449.1	287.05	Luteolin-7-*O*-glucoside
8	14.14	451.12	289.07, 99.01	Santianol-7-*O*-glucoside
9	16.28	611.15	499.1	Kaempferol-O-hexose-C-hexoside
10	16.66	595.16	433.11, 313.07	Apigenin-C-diglucoside
11	17.13	611.15	499.1	Luteolin-3,7-diglucoside
12	19.9	579.16	433.11	Apigenin-7-*O*-rutoside

### Transcriptome profiling of *H. syriacus* petals in different states

#### Overview of the transcriptome sequence

Transcriptome sequencing was performed on 15 ‘Qiansiban’ samples at 5 stages. A total of 89 GB of clean data were obtained, and the amount of clean data obtained for each sample reached 6.616 GB. The GC content ranged from 44.34 to 45.62%, and the percentage of Q30 bases ranged from 93.62 to 95.03% (Supplementary Table S2).

**Fig. 2:**
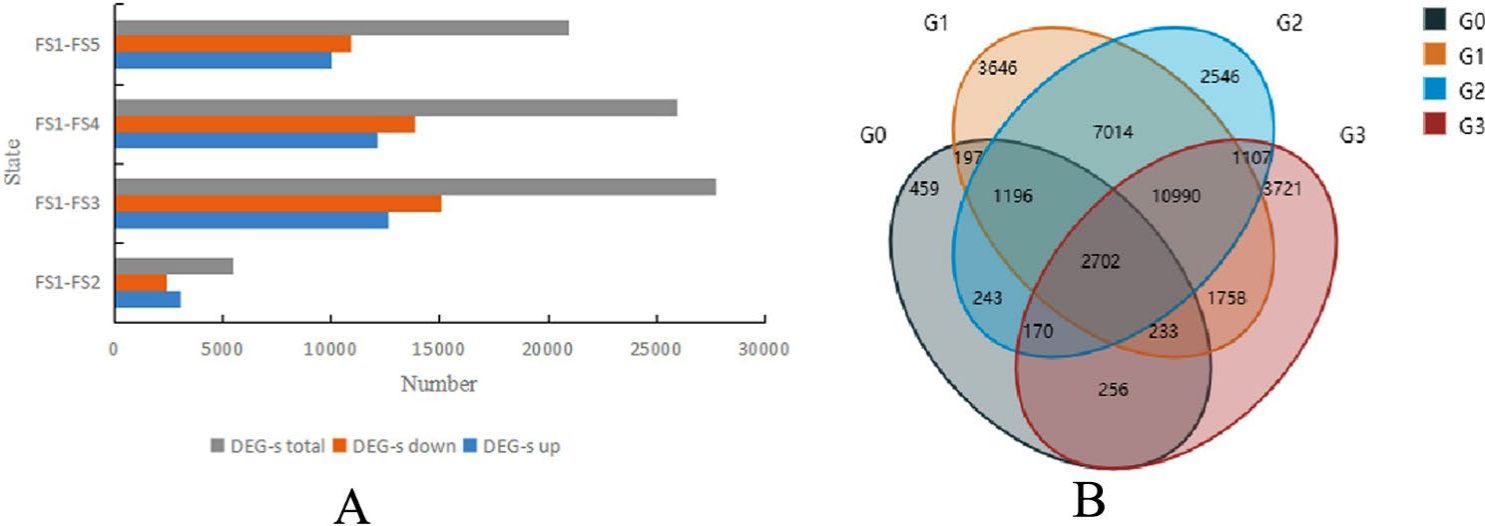
Overview of the *H. syriacus* petal transcriptome. (**A**) Statistics of DEGs between petals harvested at different developmental stages. (**B**) Venn diagram representing the common and specific DEGs identified in the three comparisons. G0: S1 vs. S2; G1: S1 vs. S3; G2: S1 vs. S4; G3: S1 vs. F5; S1–S5 represent flower development stages

### Functional annotation of new genes

The DIAMOND [17] code was used to match the newly discovered sequences with those in the NR [18], Swiss professional [19], COG [20], KOG [21], and KEGG [22] databases, and the KEGG origins and alternative results of the new sequences were obtained. InterProScan [23] used the information component of InterPro to investigate the GO Orthology [24] results of the latest genes and foreseen aminoalkanoic acid sequences of the latest genes and subsequently compared them with the Pfam [25] database victimization HMMER [26] code to obtain annotation data from the latest genes.

Based on the chosen order, the mapped reads were spliced with the NeighborTie code and were compared with the first-order annotation data. A total of 32,956 new genes were discovered, of which 23,322 were annotated. The numbers of genes annotated in different databases ranged from 4319 to 23,235, with the TrEMBL annotation having the highest number of genes and the COG annotation having the lowest number of genes (Supplementary Table S7).

### Analysis of DEGs identified in the four libraries

With the thresholds FDR < 0.01 and FC > 2, 3456 DEGs (3,053 upregulated and 403 downregulated) were identified between the FS1 and FS2 libraries. Moreover, the number of DEGs between the FS1 and FS3 libraries was the highest, with 27,736 DEGs (12,655 upregulated and 15,081 downregulated). A total of 25,968 DEGs (12,124 upregulated and 13,844 downregulated) were identified between the FS1 and FS4 libraries, and 20,937 DEGs (10,013 upregulated and 10,924 downregulated) were identified between the FS1 and FS5 libraries (Fig. 2A). There were 2,702 common DEGs among the 5 comparisons (Fig. 2B).

**Fig. 3:**
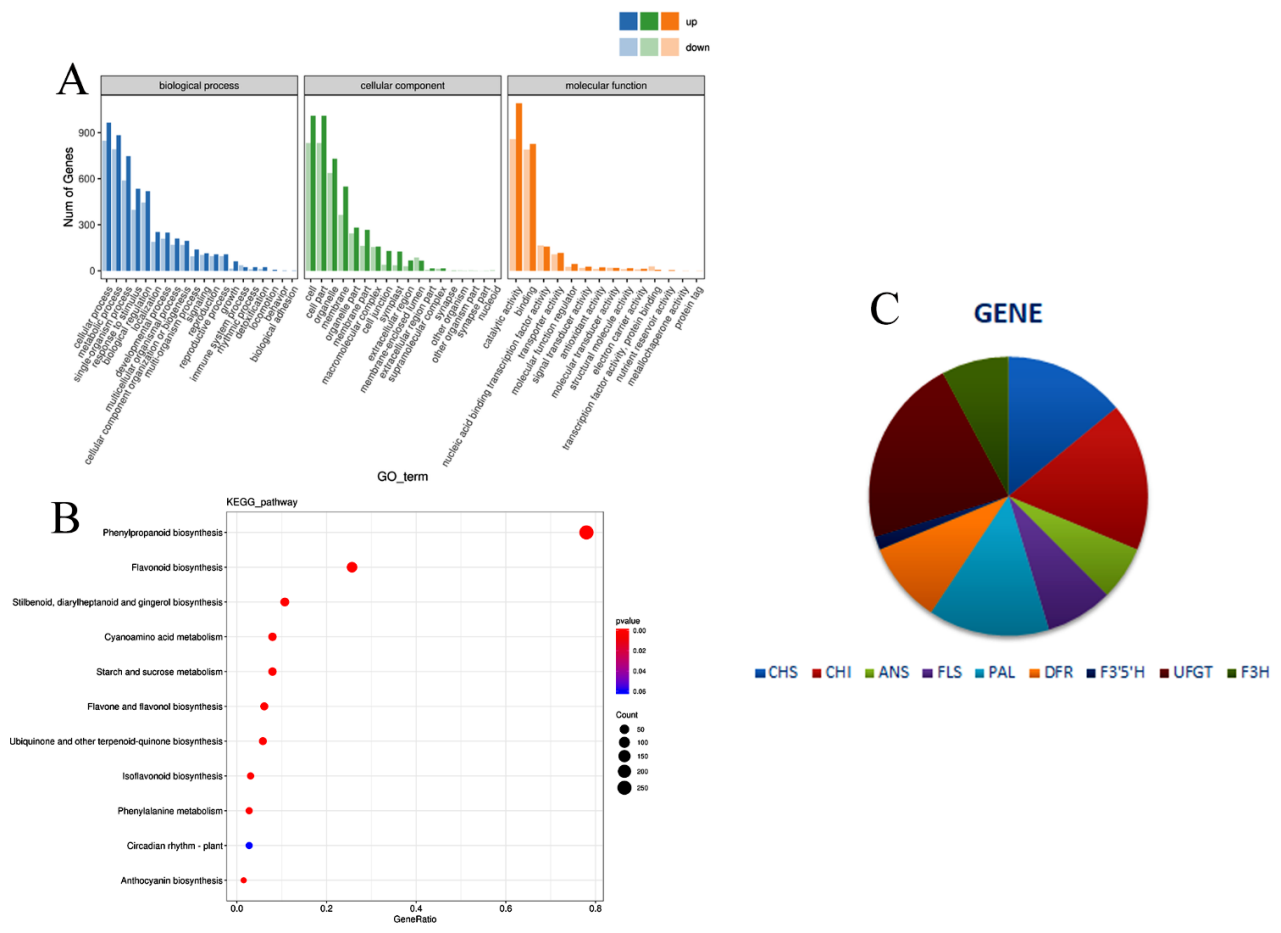
Differential gene analysis (**A**) Differential expression gene GO annotation classification statistical chart. (**B**) Metabolic pathway map of KEGG differential genes in *H. syriacus* ‘Qiansiban’. (**C**) Number of differentially expressed genes

GO enrichment analysis was also conducted on the screened differentially expressed genes, and Fig. 3A shows the top 20 pathways associated with the genes with the highest GO enrichment. The DEGs were distributed in three primary categories: biological process, cell composition, and molecular operation. The genes were enriched in cell process, biological process, and single-organism process in the biological process category; catalytic activity, binding, and transporter activity in the cell composition category; and cell, cell part, and organelle in the molecular operation category.

A differential factor KEGG metabolic pathway map was integrated into a complete metabolic pathway by classifying the DEGs [27–29]. The KEGG metabolic pathway enrichment of the ‘Qiansiban’ differential factors primarily included essential amino acid synthesis, flavonoid synthesis, cyanide metabolism, flavonoid and flavonol synthesis, essential amino acid metabolism, and anthocyanin synthesis. The metabolic pathways associated with flower colour formation included essential amino acid synthesis and metabolism, flavonoid and flavonol synthesis, and anthocyanin synthesis (Fig. 3B).

By analysing the transcriptome sequences of *H. syriacus*, combined with the KEGG annotation, 64 differentially expressed genes were obtained from the 5 stages of ‘Qiansiban’. The gene with the greatest variety was UFGT, with fourteen, and the gene with the smallest variety was F3’5’H, with one (Fig. 3C).

### Identification of structural genes involved in the flavonoid synthesis pathway

**Fig. 4:**
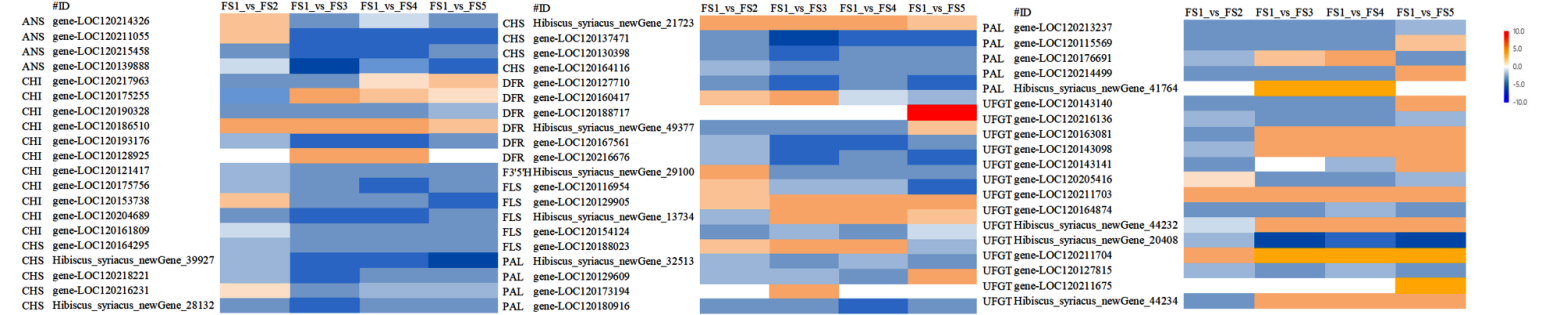
Heatmaps (log 2(fold change)) of the anthocyanin biosynthesis genes. S1 (splitting stage), S2 (early stage), S3 (initial opening period), S4 (blooming period), and S5 (final flowering stage) represent the flower development stages

**Fig. 5:**
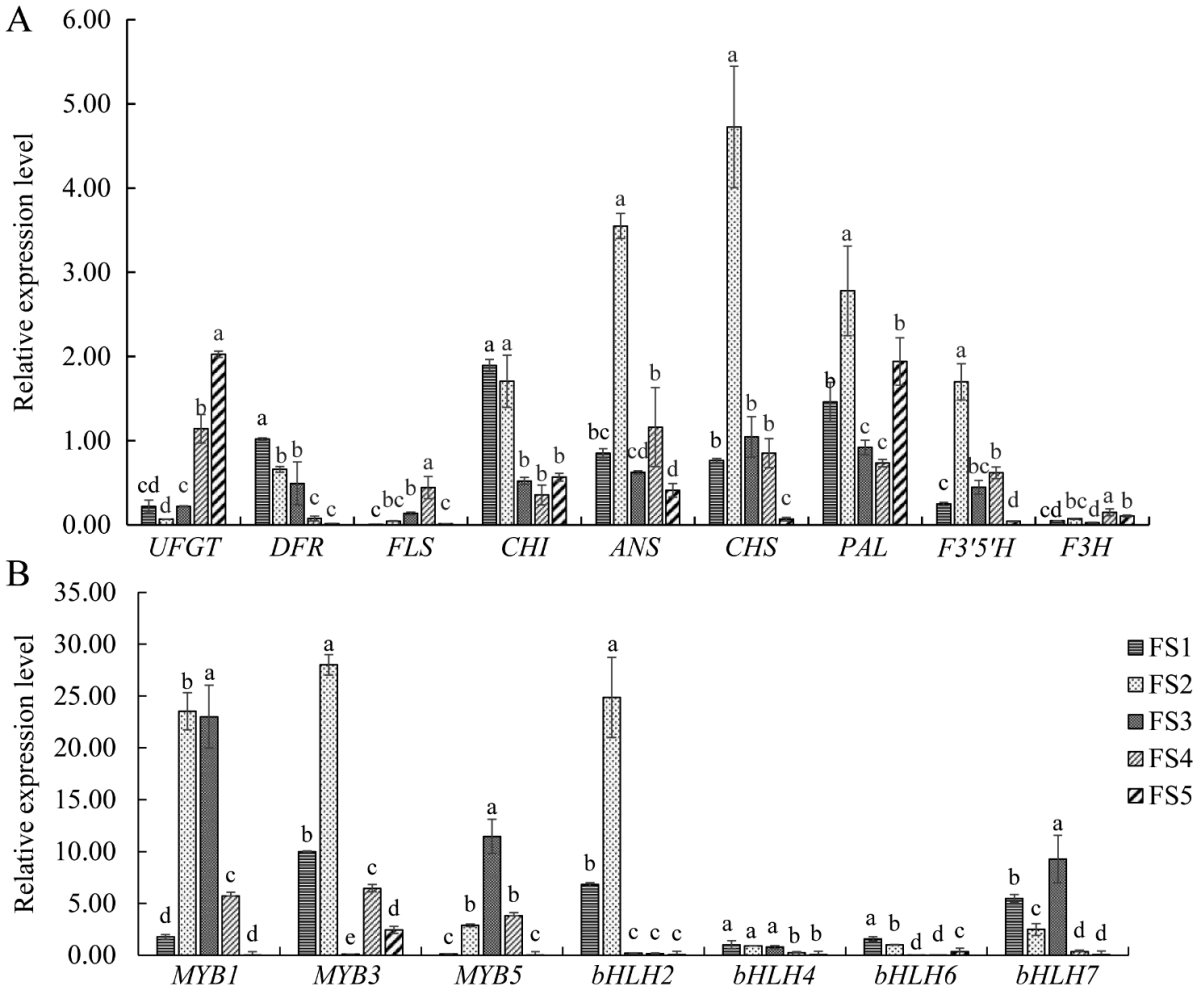
qRT‒PCR expression of anthocyanin-related genes. (**A**) The relative expression levels of the structural genes; (**B**) The relative expression levels of the transcription factors. The lowercase letters on the histogram represent differences at the 0.5 level

Transcriptome analysis revealed that sixty-four genes were considerably enriched in the flavonoid biogenesis pathway. The accumulation patterns of these anthocyanidins at different stages were consistent. The relative expression level of PAL sequentially decreased after its highest expression was reached at S2, and its expression then increased again at S5. The relative expression levels of the *CHS*1 and *ANS*1 genes also reached their highest levels in S2, with the expression of the *CHS*1 gene gradually decreasing throughout the S2–S5 stages and the expression of the *ANS*1 gene increasing in the S4 stage and then decreasing again in the S5 stage. The relative expression level of the *CHI*1 gene gradually decreased in the S1–S4 stages and then increased in the S5 stage. The relative expression level of the *FLS*1 gene increased incrementally through the S1–S4 stages and decreased in the S5 stage. The relative expression of the *DFR*1 gene decreased incrementally through the S1–S5 stages. The relative expression of the *UFGT*1 gene was the lowest in the S2 stage and increased in the S2–S5 stages. The relative expression of *F3H*, which was associated with the *F3’5’H* gene, showed an increasing decreasing trend, reaching its highest values in the S4 and S2 stages (Figs. 4 and 5, Supplementary Tables S3–S6).

According to the GO and KEGG enrichment maps constructed for the DEGs, nine structural genes were found to play key roles in the change in flower colour in ‘Qiansiban’, namely, *UFGT*1 (gene-LOC120143098), *DFR*1 (gene-LOC120216676), *CHI*1 (gene-LOC120193176), *FLS*1 (gene-LOC120188023), *ANS*1 (gene-LOC120214326), *CHS*1 (Hibiscus_syriacus_newGene_21723), *PAL*1 (Hibiscus_syriacus_newGene_32513), *F3’5’H* (Hibiscus_syriacus_newGene_29100) and *F3H* (gene-LOC120154124).

### Changes in transcription factor expression

Transcription factors such as *MYB*, *bHLH*, and *WD40 (MBW*) affect the colour of *H. syriacus* flowers by regulating the synthesis of anthocyanins. According to transcriptome sequencing and classification statistics, the expression levels of five transcription factors, namely, AP2-ERF-EFR, *MYB*, *bHLH*, *C2H2*, and *NAC* (Supplementary Figure S1), were relatively high in ‘Qiansiban’. Among them, there were 279 *MYB* transcription factors and 203 *bHLH* transcription factors (Supplementary Table S4).

Based on the GO and KEGG enrichment results, the transcription factors (TFs) encoded by the DEGs and nine structural genes were subjected to protein‒protein interaction analysis to screen for crucial TFs that control anthocyanin synthesis. The results were as follows (Supplementary Table S4): *bHLH*2 (gene-LOC120149108), *bHLH*4 (gene-LOC120171442), *bHLH*6 (gene-LOC120209233), *bHLH*7 (gene-LOC120215242), *MYB*1 (Hibiscus_syriacus_newGene_28382), *MYB*3 (gene-LOC120154943), and *MYB*5 (gene-LOC120214348) may participate in the regulation of the seven structural genes.

### qRT‒PCR analysis and validation of anthocyanin synthesis structural genes

To verify the accuracy of the RNA-Seq results, we selected seven structural genes and seven regulatory genes involved in flavonoid metabolism and analysed their relative expression levels in the five flowering stages through qRT‒PCR (Fig. 5). The qRT‒PCR results were consistent with the trends in the relative expression levels of the genes identified in the sequencing data (Supplementary Tables S3, S4), indicating that the RNA-Seq results were reliable.

**Fig. 6:**
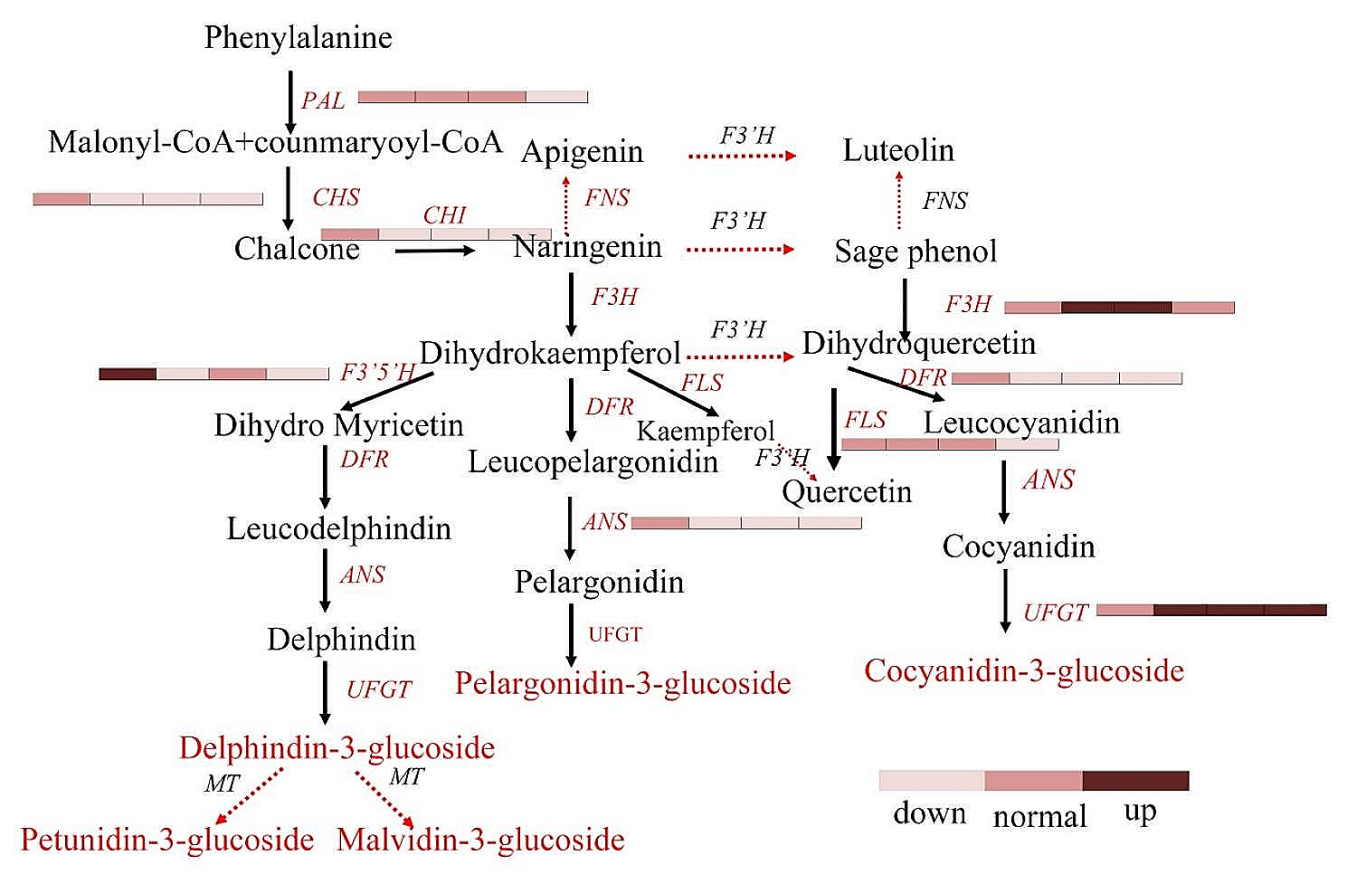
Differential regulation of anthocyanin biosynthesis in *H. syriacus* petals. The genes and metabolites that were differentially expressed and accumulated, respectively, are notated in red text. The dotted arrows/lines indicate that the genes were not found or are not known. The transcriptome and metabolome data were analysed according to the Kyoto Encyclopedia of Genes and Genomes (KEGG) metabolic pathway analysis, and a flow chart was drawn. The Kyoto Encyclopedia of Genes and Genomes has authorized the use of these images. Each of the 9 colour bars includes 4 blocks, and the 4 blocks from left to right represent the trends of change of the relative expression levels of related genes in the S2, S3, S4, and S5 stages compared with the S1 stage. The colours of the blocks from light to deep represent downregulated, normal, and upregulated relative expression, respectively. The gene name abbreviations correspond to the full names given in Supplementary Table S6

### Combined analysis of the transcriptome and metabolome

As summarized in Supplementary Table S5, *bHLH2, bHLH4*, and *bHLH6* were significantly negatively correlated with *UFGT*. *bHLH*6 and *bHLH*7 were significantly positively correlated with *PAL*, *DFR*, and *CHI*. These findings indicate that increases in the expression levels of these two *bHLH* genes will also promote the synthesis of anthocyanins. *bHLH*2 and *bHLH*4 were significantly positively correlated with *ANS*, *F3’5’H*, and *DFR*, respectively, indicating that the expression levels of some structural genes also increased with increasing flower height, thereby promoting the accumulation of anthocyanins. In terms of correlations with total anthocyanins, four *bHL*H and *MYB*3 genes were significantly positively correlated with total anthocyanins, indicating that they can also promote the generation of total anthocyanins, thereby affecting petal colour.

Figure 5 shows that the changes in the expression levels of *DFR* and *bHLH*4 were the same as the changes in the anthocyanin contents during flowering (Fig. 1), indicating that these structural genes may be induced by transcription factors to regulate the colour of ‘Qiansiban’ petals.

## Discussion

### Petal colour transition is attributed to changes in the most highly expressed anthocyanins

By using a combination of transcriptomic and metabolomic methods, the reasons for the changes in colour of ‘Qiansiban’ during flowering were investigated. The results revealed that the changes in colour of the ‘Qiansiban’ fruits were closely related to the pigment composition and contents in the petals. The composition and contents of anthocyanins change continuously during development, which in turn affects the external colour of the petals. The anthocyanin contents decreased from S1 to S5, and the colour of the petals changed from purplish red to purple (Fig. 1). Y Q et al. [30] reported that the petal colours of two peony varieties changed from red to yellow during the flowering process, mainly due to the drastic decrease in anthocyanin content. Similarly, purple jasmine (*Mirabilis jalapa* L.) flowers change from light green in the bud stage to purple‒red in the blooming stage, which is mainly related to a decrease in chlorophyll content and an increase in anthocyanin content [2]. These studies further support our conclusions. In this study, a total of five anthocyanins (Ma3G, De3G, Pe3G, Cy3G, and Pg3G) were detected in ‘Qiansiban’ (Fig. 1; Table 1), which are the same as the types of anthocyanins previously identified in *H. syriacus* petals [8]. The proportion of total anthocyanins in these plants changes constantly, and because the anthocyanins can exhibit different colours, this change in total anthocyanin content also affects petal colour.

The petals of ‘Qiansiban’ contain flavone and flavonol substances throughout the flowering process. However, their colours are not visible in the flower. Flavones and flavonols are light yellow and are speculated to be chiefly used as auxiliary pigments in *H. syriacus* petals. This phenomenon is also common in other plants, as luteolin, apigenin-7-*O*-glucoside, kaempferol-3-*O*-glucoside, and different substances are expressed at high levels in *Camellia nitidissima* C. W. Chi [31] and florist’s chrysanthemum [32].

### The regulatory effects of structural genes on the petal colour transition of *H. Syriacus*

The early biogenic genes of anthocyanins include mainly genes located upstream of the anthocyanin synthesis pathway, chiefly *PAL*, *CHS*, and *CHI*, which are responsible for encoding flavonoids and flavonols and different flavonoid synthetases. *DFR*, *ANS*, *UFGT*, and other downstream genes are mainly responsible for encoding anthocyanin synthase [33]. In this study, we conducted a transcriptomic analysis of five stages (S1, S2, S3, S4, and S5) of ‘Qiansiban’ and identified 9 genes that were significantly differentially expressed during these processes. These functional genes were significantly overexpressed in the S2 stage and contributed to the accumulation of anthocyanins. However, after the S3 stage, the expression levels of these genes decreased, leading to significant decreases in the anthocyanin contents in the later stages of development (Fig. 6). These findings are correspond with the overall changes in the expression levels of related genes in *Paeonia lactiflora* Pall [3] and *Paeonia* × *suffruticosa* Andrews [10]. The expression levels of genes in *P. lactiflora* and peony reach their maximum in the early stages of flowering and gradually decrease as their anthocyanin contents decrease throughout the entire flowering process.

*FLS* is the key factor involved in flavonol synthesis. Additionally, to aid in anthocyanin colouration, flavonols appear as a very light yellow once they are highly abundant. However, whether the expression of *FLS* causes an increase in flavonol content has yet to be verified. The *DFR* gene chiefly catalyses dihydroflavonol to synthesize colourless anthocyanins, whereas the *ANS* gene chiefly catalyses colourless anthocyanins to make coloured anthocyanins [34]. However, the high expression levels of most genes in S2, S3, and different early flowering stages promoted the synthesis of anthocyanins in the later stages. Reductions in organic phenomena within the later 2 stages resulted in a reduction in anthocyanin content, leading to changes in flower colour [1]. The analysis of *Chrysanthemum* × *morifolium* Ramat [35] and different decorative plants verified these findings.

### Relationships between transcription factors and regulatory genes

The transcription factors *MYB* and *bHLH* can affect flower colour by regulating the expression of structural genes in the anthocyanin metabolism pathway. This study identified a total of 3 *MYBs* and 4 *bHLHs*. Correlation analysis revealed that the *MYB*3 and 4 *bHLH* genes were closely related to functional genes. These genes interact with structural genes to increase or decrease their expression, thereby affecting the flower colour of ‘Qiansiban’. In recent years, studies on *Lilium davidii* var. *willmottiae* (E. H. Wilson), Raffill [36], *Prunus persica* (L.) Batsch [37], *Morus alba* L. [38], and different plants have shown that *MYBs* and *bHLHs* are key transcription factors involved in plant anthocyanin synthesis. These findings also confirm the role of these genes in ‘Qiansiban’.

Although the *MYB* and *bHLH* transcription factors are involved in anthocyanin regulation, the regulatory patterns of different transcription factors are very different. Some *MYB*s directly bind to the promoters and promote the expression of structural genes and subsequently activate the entire anthocyanin expression network [39]. This type of interaction is very common between *MYB* and genes regulating the early stages of anthocyanin biosynthesis, such as CHS, *CHI*, and *F3’H* [40]. However, some *MYB* transcription factors require the interaction of other transcription factors, such as *WD40* and *bHLH*, to induce the expression of structural genes, thereby affecting anthocyanin accumulation [41, 42]. In particular, late biosynthesis genes such as *DFR* and *UFGT* are generally regulated by the MBW-TF ternary protein complex of *MYB*-*bHLH*-*WD*40 [43]. Therefore, simply detecting the correlations between *MYB* and structural genes may not yield the desired results. This distinction may also explain why MYB5 was not significantly correlated with any of the structural genes. Niu et al. reported that the activation of the DFR promoter and the promotion of anthocyanin synthesis by MrMYB1 in *Myrica rubra* (Lour.) S. et al. are dependent on the coexpression of *bHLH* [44]. Similarly, in the absence of the *FhbHLH* cofactor, *FhMYB5* had no significant effect on activating *FhDFR3*, *FhCHI2*, *FhCHS*1, *FhF3’5’H*, or *FhF3H* in *Freesia hybrida* Klatt [45].

## Conclusion

Through metabolome and transcriptome sequencing and qRT‒PCR analysis, the colour changes of *H. syriacus* ‘Qiansiban’ petals from S1 to S5 were studied. The colour of its petals changes from red to pink‒purple and eventually to purple, which is attributed to the reduction in anthocyanin content. The anthocyanins expressed include delphinidin-3-*O*-glucoside, cyanidin-3-*O*-glucoside, petunidin-3-*O*-glucoside, pelargonidin-3-*O*-glucoside, and malvacin-3-*O*-glucoside. According to the transcriptome sequencing results, the interactions of structural genes (*DFR*, *CHI*, and *FLS*) and regulatory genes (*MYB3* and *bHLH2*, *bHLH4*, *bHLH6*, and *bHLH7*) regulates the petal colour changing process of *H.* syringae.

### Electronic supplementary material

Below is the link to the electronic supplementary material.

Supplementary Material 1

Supplementary Material 2

## Data Availability

All transcriptomic sequencing data associated with this study have been submitted to the NCBI SRA under the accession number PRJNA995230.
