# Supplementary figures and images for: Study on the causes of changes in colour during Hibiscus syriacus flowering based on transcriptome and metabolome analyses
Source: BMC Plant Biol. 2024 May 21;24:431. doi: 10.1186/s12870-024-05142-0 (PMC11107057; doi:10.1186/s12870-024-05142-0)

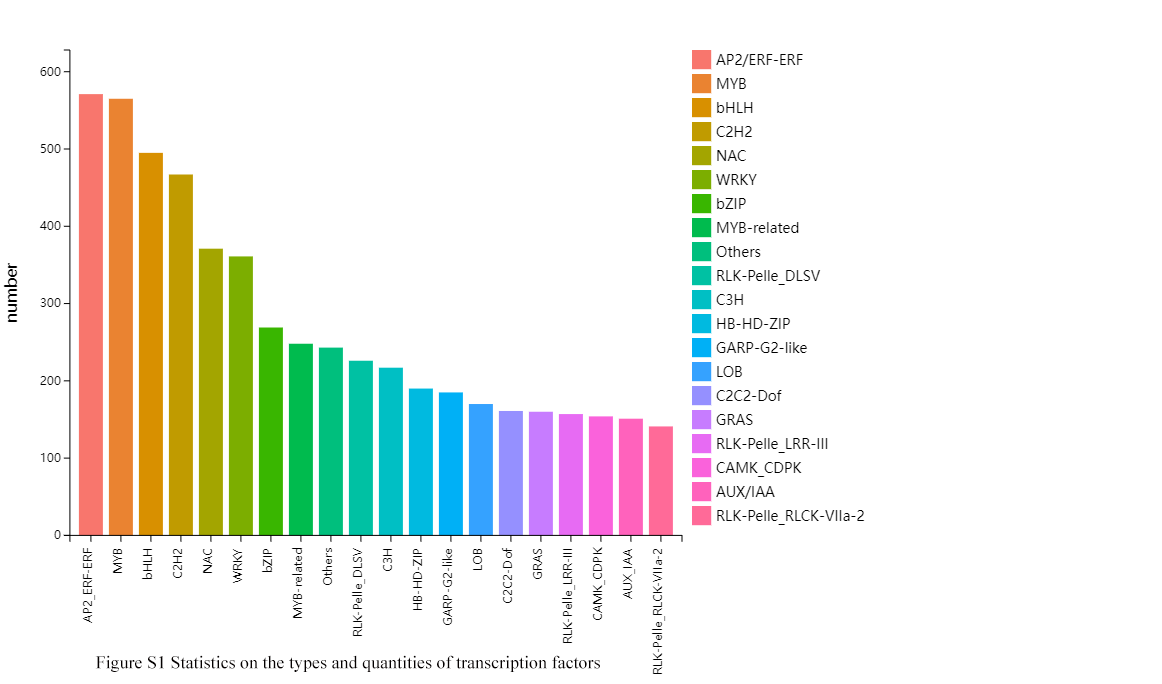

Supplement: Supplementary file 2 — Supplementary Material 2 [file 12870_2024_5142_MOESM2_ESM.tif]
